# Genetics and genomics in Thailand: challenges and opportunities

**DOI:** 10.1002/mgg3.83

**Published:** 2014-05-14

**Authors:** Vorasuk Shotelersuk, Chanin Limwongse, Surakameth Mahasirimongkol

**Affiliations:** 1 Center of Excellence for Medical Genetics, Department of Pediatrics, Faculty of Medicine, Chulalongkorn UniversityBangkok, 10330, Thailand; 2 Excellence Center for Medical Genetics, King Chulalongkorn Memorial Hospital, the Thai Red Cross SocietyBangkok, 10330, Thailand; 3 Departments Medicine and Research and Development, Faculty of Medicine Siriraj Hospital, Mahidol UniversityBangkok, 10700, Thailand; 4 Medical Genetic Centre, Medical Life Sciences Institute, Department of Medical Sciences, Ministry of Public HealthNonthaburi, 11000, Thailand


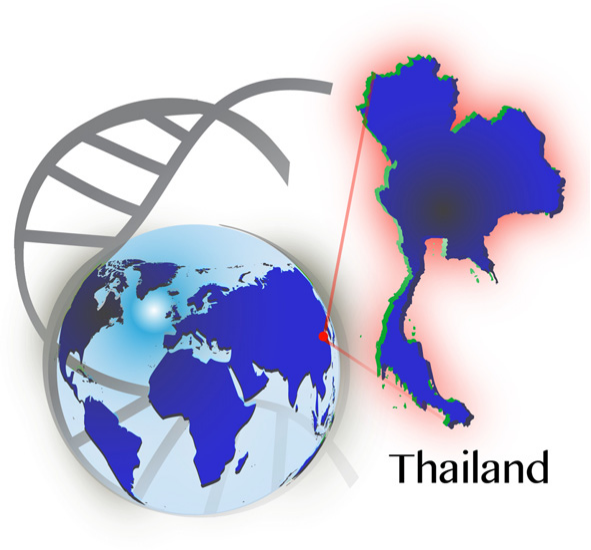


## Thailand: A Developing Middle Income Country

**Figure 1:**
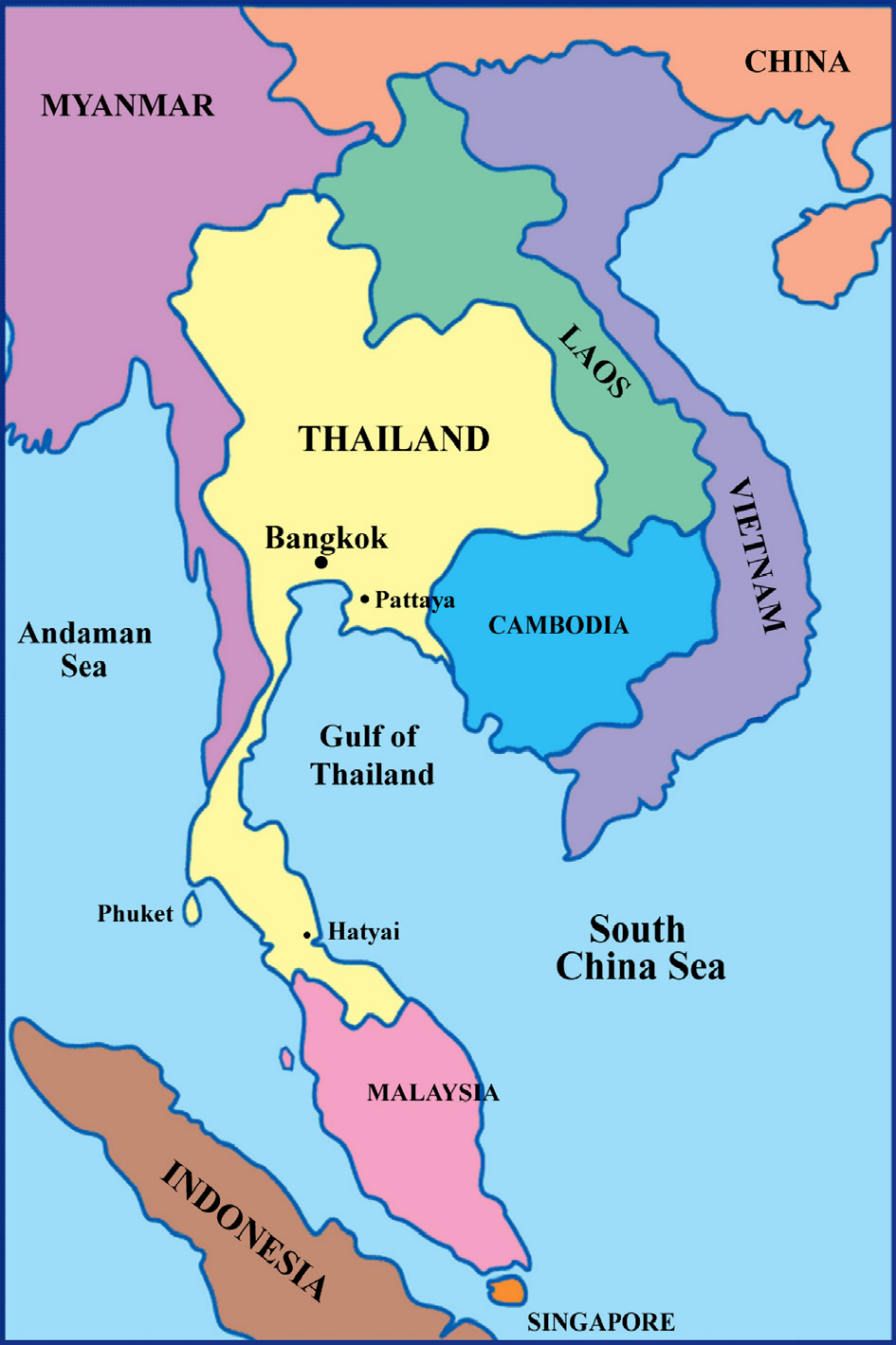
Map of Thailand.

Thailand's history goes back more than 700 years, and since 1782, Bangkok has been the capital. The population of Thailand in 2013 was 69.52 million, ranked 20th in the world (http://www.worldpopulationstatistics.com/). More than 14 million (or 22% of the total population) live in the Bangkok Metropolitan Region, located in Central Thailand (http://worldpopulationreview.com/countries/thailand-population/) (Fig. 1). The original Thai were thought to have descended from the Altai Mountain region in the South of China. More recent genetic evidence suggests that the origin of Thai was from India (Abdulla et al. 2009). Seventy-five percent of all people in Thailand are ethnic Thais. From about 1850 to the end of the World War II, there was a large and steady wave of Chinese immigration to Thailand. Currently, around 14% of the population has Chinese origin and the remaining 11% is made up of various other groups (http://en.wikipedia.org/wiki/Demographics_of_Thailand). Subsequent generations of Chinese in Thailand have Thai names, speak Thai, consider themselves Thai, and have married original Thai. Therefore, it is now very difficult to distinguish the original Thai from Chinese Thai. Although not strictly prohibited, consanguineous marriages are strongly discouraged in both original Thai and Thai–Chinese cultures.

Thailand's gross domestic product (GDP) was 366 billion U.S. dollars (USD), ranked 21st in the world, and is grouped in the upper middle income category by the World Bank. (http://data.worldbank.org/country/thailand). The politics of Thailand are a constitutional monarchy, whereby the Prime Minister is the head of government and a hereditary monarch is head of state, and the judiciary branch is independent of the executive and the legislative branches. Since 2001, populist policies have been implemented including a universal healthcare scheme, which has tremendously impacted the medical system and genetic service in Thailand.

## Medical and Genetic Services

### Health, medical service in general

Thailand has had a successful history of health improvement with a life expectancy of 71 years for males and 78 years for females, and with a reduction in infant mortality from 68 per 1000 in 1970 to 13 per 1000 today (Statistical Thailand, 2013; http://bps.ops.moph.go.th/).

The public health system in Thailand had been transformed since the adoption of the National Health Security Act in 2002 (Tangcharoensathien et al. 2011) with the National Health Security Office (NHSO) financing the health system and the Ministry of Public Health (MOPH) providing health services. As a research institute within the MOPH, the Health Intervention and Technology Assessment Program (HITAP) provides evidence-based economic healthcare policy evaluations which determines covered services (Mohara et al. 2012).

Three reimbursement systems are in place, the Universal Health Coverage Scheme, the social security system, and the government officer reimbursement plan, resulting in more than 96% of the population having health insurance that covers preventive and treatment costs including genetic services. Unfortunately, some standard tests are not currently covered, for example, molecular testing for possible carriers of X-linked disorders.

In 2008, 1226 hospitals (954 public and 272 private) with 125,866 beds were available to provide in-patients services. Both the private and public sector healthcare workers receive training funding from the human resource development effort of the MOPH (Tangcharoensathien et al. 2013).

### Genetics services and testing

During the 1970s, specialists graduating from training programs in either the United Kingdom (U.K.) or the United States (U.S.A.) established clinical genetic services in Thailand in both hematology and pediatric departments. In the 1980s, cytogenetic services began in Thai university hospitals as laboratory specialist received training abroad, and since the 1980s, governmental cytogenetic laboratories have become available throughout the country. On a much smaller scale, private sector laboratories also provide genetic services.

Molecular genetic testing for thalassemia became available 30 years ago and was the first available genetic test excluding karyotypes. With its abundance of thalassemia, Thailand has always been one of the world leaders for advancing research in hemoglobinopathies (Wasi et al. 1964, 1967; Clegg et al. 1968; Na-Nakorn et al. 1969). Expansion of genetic testing to other disorders began around 1990 with evaluations for dystrophinopathy, autosomal dominant polycystic kidney disease (ADPKD), and spinocerebellar ataxia type 3.

Since 1999, a continued presence of board certified geneticists has been available in Thailand. Currently, there are 22 practicing clinical geneticists, and 11 are diplomates of the American Board of Medical Genetics. With better testing including fluorescence in situ hybridization and cancer cytogenetics, and more practicing genetic physicians, the specialty of genetics has gained more acceptance and referrals and consultations have grown.

Prenatal diagnosis for Mendelian diseases and preimplantation genetic diagnosis (PGD) have been made available over the past 20 years with test panels now comparable to most developed countries. Noninvasive prenatal fetal trisomy (NIFTY) testing as well as rapid invasive diagnostic methods for common aneuploidy are available as send out tests to out of country laboratories. Two university-based laboratories and one private laboratory are currently providing PGD services for a few Mendelian diseases including thalassemia (Piyamongkol et al. 2006).

Currently, ten governmental and seven private cytogenetic laboratories provide karyotyping service, four university-based molecular laboratories carry out DNA testing for Mendelian disorder, and a single university laboratory provides mitochondrial DNA testing. Five governmental laboratories handle pharmacogenetic testing for HLA-B*1502, which is a susceptibility allele for Stevens–Johnson syndrome in-patients treated with carbamazepine, and three other laboratories provide other pharmacogenetic testing. For cancer-related pharmacogenetic testing, two governmental laboratories offer testing such as KRAS mutation status in candidates for anti-epidermal growth factor receptor therapy. There are three biochemical genetics laboratories located in university hospitals in Bangkok providing urine organic acid analysis and plasma amino acid analysis (Shotelersuk et al. 2000). Although enzymatic assays of various enzymes were desired, sufficient financial support was not available and therefore, most inherited metabolic disorders are diagnosed by molecular testing (Champattanachai et al. 2003; Shotelersuk et al. 2004; Tammachote et al. 2009, 2010, 2012, 2013; Amarinthnukrowh et al. 2010; Prommajan et al. 2011; Vatanavicharn et al. 2012). If enzymatic assays are needed, they are sent out to other countries.

Next generation sequencing (NGS) technologies for research have been available in Thailand for a few years with at least five institutes in Thailand possessing NGS technology. NGS services are currently limited by a deficiency in well-trained bioinformaticians.

With all the progress in genetic services over the last 40 years, there are still only 22 clinical geneticists available to cover 69 million people, and much of their time is spent as genetic laboratory supervising. There are no genetic counselors in Thailand, adding to the manpower shortage. A local postgraduate fellowship training program for medical (pediatric) geneticist is currently approved for 2014 with the hope of supplementing the next generation of graduates to work in this field.

### Coverage of genetic testing under the universal healthcare scheme

#### National neonatal screening operation centre

Neonatal screening programs are administered by two organizations, the national neonatal screening operation center of MOPH that operates in all provinces and metabolic genetic center of Mahidol University located in Bangkok. Since 1996, these centers have provided coverage for 95% of all newborns for congenital hypothyroidism (Charoensiriwatana et al. 2003) and phenylketonuria (Pangkanon et al. 2009)(Charoensiriwatana et al. 2008). Other neonatal screening for inborn errors of metabolism besides these two diseases is not funded.

#### Thalassemia

The national program for prevention and control of thalassemia has been a 30-year struggle due to the mutation heterogeneity and complexity, the differential access to health care among regions, and the inconsistent governmental funding. Twenty years ago, Thailand established a national prevention and control policy with two objectives: to offer high-quality care for the affected (Abdulla et al. 2009) and to provide universal screening and counseling for couples at risk of having a child with severe thalassemia (Tangcharoensathien et al. 2011). With the academic support of the Thalassemia Foundation of Thailand, the Ministry of Health has been the leader in the effort to prevent and treat thalassemia. Since 2003, a nationwide screening strategy has been implemented in prenatal clinics with reflexive spouse testing. For identified couples at risk, counseling and prenatal diagnosis is offered, and pregnancy termination offered if the fetus is affected with one of the three severe forms of alpha or beta thalassemia. Specific mutation testing for beta thalassemia was introduced in 2010 in order to predict the severity of beta thalassemia/Hb E disease, the most prevalent severe form. Couples found to be at risk of mild nontransfusion-dependent thalassemia are currently encouraged to continue their pregnancy. From the treatment standpoint, major advances in the past 10 years include maintaining an adequate blood supply for transfusions, making available local-made oral iron chelating medication, and continuous training of healthcare personnel specializing in the care of thalassemia major. Due to these major achievements, there are almost no cases of Hb Bart's hydrops fetalis and the majority of pregnancies with DNA proven major beta thalassemia are terminated in the second trimester.

#### Pharmacogenetics

Pharmacogenetics testing has been implemented nationwide and in 2013, university- based medical centers including Chulalongkorn University, Mahidol University, KhonKaen University, and Prince of Songkla University established pharmacogenetics testing services. Recently, HLA-B*1502 testing used for prevention of severe cutaneous adverse reactions (SCAR) from carbamazepine was proven to be cost-effective in Thailand which is the reason this makes up most of the pharmacogenetic testing (Rattanavipapong et al. 2013). The HLA genotyping laboratories have been compelled to extend their capacity as Thailand is among the top countries in number of SCAR cases from The *Uppsala Monitoring Centre* (UMC) and pilot testing for HLA-B*1502 has been launched in Bangkok through supports of NHSO's Bangkok Office.

#### Outreach clinics

Since resources are mostly pooled in Bangkok and a few other big cities, many patients in rural areas do not have access to medical and genetics services. Many outreach clinics have been started to help alleviate this problem. As an example, the Smart Smile and Speech Project was initiated by the Thai Red Cross Society to properly and holistically treat all children with oral clefts born in Thailand since 2005 is one of the very successful projects.

#### Birth Defect Registry and folate

The Thailand Birth Defects Registry which provides preventive measures and an educational program was developed in 2008 to decrease the incidence of birth defects and to provide care for children with birth defects in a holistic manner. Care maps of five birth defects – Down syndrome, neural tube defects, cleft lip/palate, limb defects, and Duchenne muscular dystrophy – were established (http://birthdefects.nhso.go.th/BirthDefects/login.xhtml).

It has been widely accepted that periconceptional consumption of folic acid (FA) can prevent many congenital anomalies. In 2003, a survey of 383 pregnant Thai women showed 23% knew that FA helped to prevent birth defects, 3% knew that FA should be taken before pregnancy, and only 0.3% reported taking FA before pregnancy (Vilaiphan et al. 2007). The Thai Government Pharmaceutical Organization has produced Triferdine (ferrous fumarate 185 mg + iodine 0.15 mg + folic acid 0.4 mg) for pregnant women; however, FA has not been produced for women of reproductive age and there have been no government policies for educational campaigns or food fortification with FA.

## Reproductive Law

The current law permits abortion only in cases where continuing the pregnancy would jeopardize the mother's life and for rape and does not allow termination of pregnancy at any stages no matter how severe is the disease the fetus carries. Several attempts have been made to modify the law; however, Thais are 99% Buddhists and Buddhism considers conception as the beginning of life thus making termination an unfavorable idea among both pregnant couples and obstetricians alike. Nonetheless, some modification of the medical council level regulation has been put into place that allows a termination of pregnancy based on maternal mental health issues. This notion paved the way for a plausible reason for abortion under the condition that the mother could be considered to have a jeopardized mental status by knowing that her fetus was affected with a serious genetic disease. Hospital-based abortion committee could then offer inpatient abortion for pregnancy with a proven serious genetic disorder.

Medical genetic testing is regulated by the national Food and Drug Administration (FDA); however, the direct-to-consumer genetic tests are regulated by consumer law and such testing is currently not included under the jurisdiction of the national FDA. It will be interesting to see how Thai society will cope with the influx of personalized medicine as this type of testing becomes more available. There is also currently no law to prevent discrimination based on genetic status such as the Genetic Information Nondiscrimination Act (GINA) in the United States.

## Teaching

Medical school curriculum has included medical genetics as one of the required courses for more than 20 years. However, due to the shortage of medical genetic specialists, these courses are often not given by geneticists. Biologists, medical technologists, and clinical pathologists in some universities have been responsible for such teaching. With different types of curriculum, medical genetics is generally offered either as a week- or month-long program during preclinical rotations.

Clinical genetics has not been recognized as a specialty by the Medical Association of Thailand. However, a formal training program for pediatricians to become medical geneticists will be launched in the academic year of 2014, under the Royal College of Pediatricians. Also as noted above, there are no training programs for genetic counselors.

Molecular genetics is a well-established field in Thailand. There are hundreds of scientists using molecular techniques for research. However, cytogeneticists and biochemical geneticists are extremely insufficient.

## Research “Opportunities to Study Tropical Genetic Diseases in Thailand”

Thailand harbors rare genetic diseases, unique to Thailand, which have contributed to the international knowledge on diseases such as benign adult familial myoclonic epilepsy (Yeetong et al. 2013). Furthermore, clinical features of the same single gene disorders but with different ethnic background may have a different phenotype (Shotelersuk 2003). In addition to rare diseases, Thailand has a number of common genetic diseases including thalassemia, the most common Mendelian disease in Thailand. Mutation carriers were estimated at 30–40% of the Thai population. In addition to thalassemia alleles, HLA-B*1502 is found in about 9% of the Thai population making Carbamazepine and phenytoin-induced Stevens–Johnson syndrome more common in Thailand than any other place in the world (Locharernkul et al. 2008), and this association has helped identify carbamazepine-induced hypersensitivity reactions in Europeans associated with HLA-A*3101 (McCormack et al. 2011).

Many studies on genetic factors of tropical infectious diseases have been performed here, including cholangiocarcinoma which has its highest prevalence in KhonKhan, a city in northeastern Thailand. *Opisthorchis viverini* has long been found to be a risk factor for cholangiocarcinoma and recent genetic studies provide insight into the mutational landscape, which may lead to specific-targeted therapy (Ong et al. 2012). A study of dengue fever, a common tropical disease in Thailand, showed a variant in *CD209* having a crucial role in dengue pathogenesis which may allow for preventive strategies (Sakuntabhai et al. 2005).

**Figure 2:**
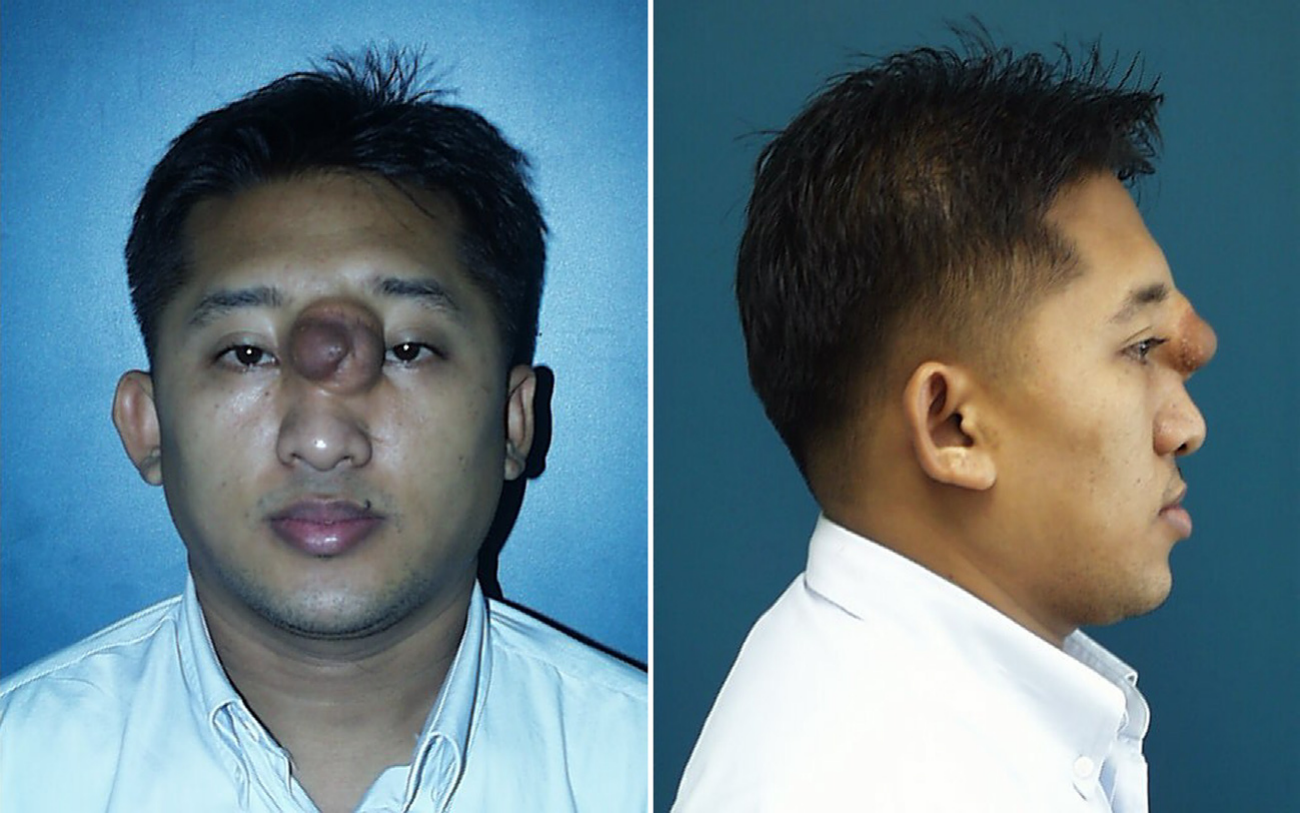
Clinical features of frontoethmoidal encephalomeningocele (FEEM), a birth defect which is more common in Thailand than other parts of the world.

The underlying causes of some common diseases in Thailand are still elusive. As an example, frontoethmoidal encephalomeningocele (FEEM), a type of neural tube defect, characterized by a congenital bone defect of the anterior cranium between the frontal and ethmoidal bones resulting in herniation of meninges and brain tissues (Fig. 2), has a unique geographical distribution. FEEM is much more common in Southeast Asia, with an approximate prevalence of 1 in 6000. Risk factors associated with FEEM include low socioeconomic status, advanced maternal age, and a long interpregnancy interval, most likely making this a multifactorial complex disease (Suphapeetiporn et al. 2008).

Oral clefts are common in Thailand and genetic studies have demonstrated many genetic susceptibility loci and new mutations (Shotelersuk et al. 2003; Srichomthong et al. 2005, 2013; Tongkobpetch et al. 2006, 2008; Suphapeetiporn et al. 2007; Rattanasopha et al. 2012), including *p63* which causes an isolated cleft lip (Leoyklang et al. 2006), and *SATB2* which causes a unique dysmorphic syndrome with intellectual deficit, *SATB2*-associated syndrome (SAS) (Leoyklang et al. 2007, 2013).

Genetic diseases with a higher prevalence in Thailand compared to other parts of the world are a compelling reason to conduct research here. Unfortunately, there are limitations including inadequate budget, insufficient advanced technologies and knowledgeable personnel in bioinformatics, and inefficient supporting systems. However, we are optimistic that collaborations between local and foreign researchers will lead to better care of patients both in Thailand and globally.
